# Dietary dihydroquercetin alleviates age-related cognitive impairment in association with modulation of apoptosis and pyroptosis pathways

**DOI:** 10.3389/fphar.2026.1744898

**Published:** 2026-02-03

**Authors:** Yongchang Zeng, Dandan Xu, Xinping Li, Qianqian Jiang, Shaoyu Liang

**Affiliations:** 1 The Affiliated TCM Hospital of Guangzhou Medical University, Guangzhou, China; 2 The First Affiliated Hospital of Shenzhen University, Shenzhen, China

**Keywords:** apoptosis, cognitive impairment, dihydroquercetin, neuroinflammation, pyroptosis

## Abstract

With the intensification of population aging, age-related cognitive decline has emerged as a significant global health issue. Neuroinflammation, neuronal apoptosis, and pyroptosis have been identified as key factors in neuronal loss, a hallmark of age-related neurodegenerative conditions. Dihydroquercetin (DHQ), a natural flavonoid and a new food resource, demonstrates remarkable antioxidant and anti-inflammatory properties; however, its capacity to mitigate cognitive impairment in context of apoptosis and pyroptosis remains to be fully elucidated. In this study, an ageing rat modelling by intraperitoneal injection of D-galactose was utilised to assess the therapeutic potential of DHQ. A comprehensive analytical technique, incorporating behavioural tests, TCM symptom scoring, histopathological evaluation (Hematoxylin-eosin staining and Nissl staining), ELISA, Western blotting, and molecular docking analysis, was performed to investigate its pharmacodynamic effects and potential mechanisms. DHQ supplementation significantly alleviated D-gal-induced aging phenotypes and improved learning and memory function in the Morris water maze test. Histopathological examinations indicated that DHQ mitigated neuronal damage and loss in the hippocampus whilst concomitantly increasing the number of Nissl bodies. Furthermore, DHQ administration suppressed neuroinflammation in hippocampus, as indicated by decreased levels of TNF-α, IL-1β, and IL-6. At the molecular level, DHQ treatment was associated with altered expression of proteins involved in apoptosis, specifically increasing the expression of the anti-apoptotic protein BCL2 and reducing the expression of the pro-apoptotic proteins BAX and CASP3. Crucially, DHQ supplementation significantly suppressed the activation of the TLR4/NF-κB/NLRP3/CASP1 pyroptosis-related pathway, as evidenced by decreased protein expression of TLR4, NF-κB p65, p-NF-κB p65, NLRP3, CASP1, and mature IL-1β. Molecular docking predictions suggested potential binding interactions between DHQ and key targets within both apoptotic and pyroptotic pathways. DHQ exerts a mitigating effect on age-associated cognitive impairment, possibly through its association with reduced neuroinflammation and dual modulation of neuronal apoptosis and the NLRP3 inflammasome-mediated pyroptosis pathway. This positions DHQ as a promising candidate for further investigation as a therapeutic agent or dietary supplement in ageing-related neurodegenerative conditions.

## Introduction

The global demographic landscape is undergoing a profound shift towards an aging population, accompanied by a surge in age-associated neurodegenerative disorders. Among these, age-related cognitive impairment, encompassing a range of conditions from mild cognitive decline to severe dementia, including Alzheimer’s disease, represents a considerable challenge to public health systems worldwide. Epidemiological studies indicate that more than 50 million people are diagnosed with dementia, with numbers projected to triple by 2050, creating an unsustainable socioeconomic burden (Gauthier et al., 2021; Zhang et al., 2021). Despite extensive research, the precise mechanisms driving cognitive impairment remain unclear, and the complex biomolecular networks associated with its progression are still ambiguous, leading to extremely limited therapeutic options against cognitive decline. Current pharmacological interventions, predominantly acetylcholinesterase inhibitors (e.g., donepezil) and NMDA receptor antagonists (e.g., memantine), yield only modest symptom relief without halting or reversing the underlying pathological progression (Passeri et al., 2022). Furthermore, their practical application is frequently undermined by a relatively low level of effectiveness and notable side effects, including gastrointestinal disturbances and cardiovascular issues (Cummings et al., 2019). In view of the complex pathological and physiological processes involved in cognitive impairment, there is an urgent need to identify effective pharmaceutical interventions that demonstrate both safety and multi-targeted efficacy, with the objective of decelerating the progression of the condition.

A substantial body of evidence indicates that, in addition to amyloid-β (Aβ) plaques, neurofibrillary tangles, and synaptic loss, neuroinflammation is now recognized as a critical driver of cognitive impairment progression, rather than merely a bystander effect (Heneka et al., 2015). The activation of microglia, triggered by damage-associated molecular patterns (DAMPs), initiates a self-perpetuating cycle of inflammation, largely mediated by the Toll-like receptor 4 (TLR4)/NF-κB signaling axis. TLR4 activation leads to NF-κB nuclear translocation and the transcription of pivotal pro-inflammatory cytokines, including IL-1β and IL-18 (Liu et al., 2020; Squillace and Salvemini, 2022; Pascual et al., 2021). Crucially, the TLR4/NF-κB pathway functions as a pivotal upstream regulator of NLRP3 inflammasome activation, thereby establishing a self-sustaining “neuroinflammation-neurodegeneration vicious cycle.” NF-κB primes the NLRP3 inflammasome by upregulating the components of the NLRP3 inflammasome, including NLRP3, ASC, and procaspase-1 (Yang et al., 2020). Subsequent inflammasome assembly triggers caspase-1 autocleavage, activating IL-1β/IL-18 maturation and GSDMD cleavage. The liberation of GSDMD-NT results in the formation of membrane pores, which in turn induce microglial pyroptosis (Kelley et al., 2019). Furthermore, the release of both IL-1β and TNFα induces disruption of the blood–brain barrier, resulting in the infiltration of peripheral immune cells into the brain (Shen, 2012). Of equal importance is the fact that inhibition of TLR4/NF-κB results in a reduction of NLRP3 activation, CASP1 cleavage, and IL-1β maturation, thereby suppressing pyroptosis and neuroinflammation (Cui et al., 2020; He et al., 2020). In addition, chronic neuroinflammation directly instigates neuronal apoptosis. Pro-inflammatory cytokines activate extrinsic apoptotic pathways and disrupt mitochondrial integrity, leading to a dysregulation of BCL2 family proteins (e.g., BAX) and the activation of executioner caspases such as CASP3 (Rajesh and Kanneganti, 2022; Burguillos et al., 2011; Kaushal et al., 2015; Yap, et al., 2019). Therefore, the TLR4/NF-κB/NLRP3/CASP1 axis represents a promising therapeutic target, the modulation of which has the potential to disrupt neuroinflammation, pyroptosis, and apoptosis simultaneously.

Dihydroquercetin (DHQ, also known as taxifolin), is a natural bioactive flavonoid abundant in Pinaceae plants, such as *Larch* and *Douglas fir*. In 2021, the National Health Commission of China formally recognised it as a novel food raw material, thereby permitting its use in both food additives and functional foods (Yang et al., 2023; Sunil and Xu, 2019). Dut to its multifaceted pharmacological effects, including antioxidant [Ahiskali et al., 2019; Xie et al., 2017), anti-inflammatory (Zhan et al., 2021), anti-cancer (Li et al., 2019), hepatoprotective (Chen et al., 2018), and anti-glycation properties (Harris et al., 2011), DHQ has attracted mounting interest as a potential therapeutic agent for a range of diseases, including cancer, dyslipidaemia, cardiovascular diseases, viral hepatitis and neurodegenerative disorders. It is important to note that DHQ has been observed to traverse the blood-brain barrier, thus indicating the potential for neuroprotective effects via inhibition of Aβ fibril formation and ROS production, improvement of cerebral blood flow, suppression of inflammation, increase of calcium concentration, and mitigation of glutamate levels (Inoue et al., 2019; Yang et al., 2023; Turovskaya et al., 2019). Though a mounting previous studies have demonstrated DHQ’s capacity to modulate the NF-κB pathway and NLRP3 inflammasome in rotenone-induced Parkinsonism (Akinmoladun et al., 2022), alcoholic liver steatosis (Zhang et al., 2018) or diabetic nephropathy (Ding et al., 2018), it should be noted that these studies were conducted in distinct pathological models. However, its precise mechanisms of action in brain ageing, particularly concerning the intricate interplay and concurrent regulation of neuroinflammation, neuronal apoptosis, and NLRP3 inflammasome-mediated pyroptosis in the hippocampus - a brain region critically vulnerable in age-related cognitive decline - remain poorly defined. The specific effects of DHQ on the TLR4/NF-κB/NLRP3/CASP1 signaling axis, a central hub integrating inflammatory and pyroptotic signals, and its simultaneous impact on the BCL2/BAX/CASP3-mediated apoptotic pathway within the ageing hippocampus have yet to be elucidated.

**FIGURE 1:**
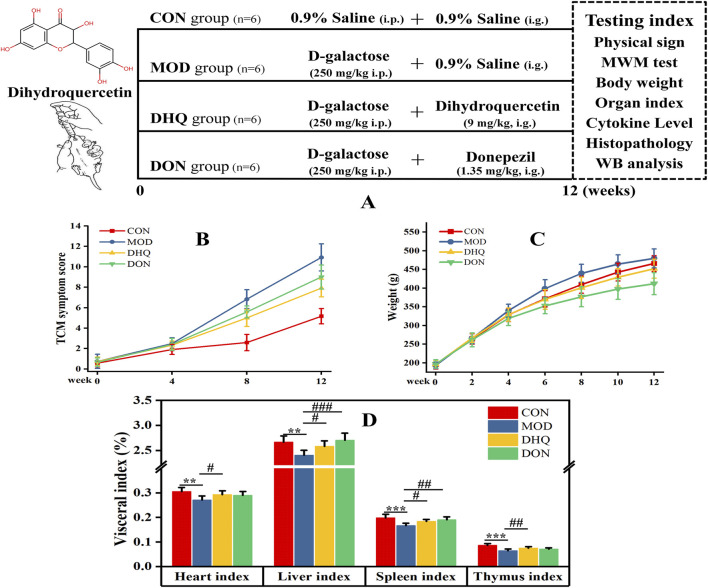
The outline of the experimental procedure **(A)**, and the effects of DHQ on TCM symptom score **(B)**, weight **(C)**, visceral index **(D)** in D-galactose-induced aging rat model. Data represented as mean ± S.D. (n = 6).

In the present study, we employed an ageing rat model induced by D-galactose to analyse the therapeutic effect of DHQ against cognitive impairment on cytokine levels and histopathological changes in brain tissue, particularly with regard to the expression of key proteins in neuronal apoptosis and NLRP3 inflammasome-mediated pyroptosis. Furthermore, utilising computational pharmacology, a molecular docking analysis was conducted to predict the direct binding affinities of DHQ with multiple targets within the aforementioned pathway. As illustrated above, a comprehensive investigation was conducted to elucidate the association between DHQ supplementation and the potent modulation of the interconnected pathways comprising neuroinflammation, apoptosis, and pyroptosis, thereby providing a robust scientific basis for the utilisation of DHQ or DHQ-rich supplements as a nutritional strategy to delay cognitive aging and mitigate the risk of age-related neurodegenerative diseases. The technological roadmap is illustrated in Figure 1A.

## Materials and methods

### Chemicals and reagents

D-galactose (purity ≥99%) and donepezil hydrochloride (purity ≥98%) were purchased from Aladdin biochemical technology Inc. (Shanghai, China). Dihydroquercetin (purity ≥98%) was purchased from Xi’an Benfeng Biotechnology Co., Ltd (Xi’an China). RIPA buffer was obtained from NCM biotech Co., Ltd. (Suzhou, China). ELISA kits for IL-6, IL-1β and TNFα were supplied by Boster biological technology Co., Ltd. (Nanjing, China). The primary antibodies for TLR4, NF-κB p65, IL-1β, CASP1, NLRP3, Bax, and Bcl2 were purchased from Proteintech group, Inc. (Wuhan, China). The primary antibodies for phospho-NF-κB p65 was obtained from Affinity bioscience Co., Ltd. (Jiangsu, China). The primary antibodies for CASP3, beta-actin, and horseradish peroxidase (HRP)-conjugated affinity pure goat anti-rabbit IgG were purchased from Boster biological technology Co., Ltd. (Nanjing, China).

### Animals and drug treatment protocols

Male Sprague-Dawley rats (180–220 g, 8 weeks old) were obtained from Zhuhai Baishitong Biotechnology Co. Ltd. (No.SCXK(Yue)2020-0051). The animals were cultivated under specific pathogen-free conditions as follows: 25 °C ± 3 °C temperature, 55% ± 10% relative humidity, a 12-h light/dark cycle, and free access to food and water.

After 1 week acclimation, SD rats were randomly divided into the four groups, with six rats per group: (i) control group (CON, 0.9% saline i.p.+0.9% saline, i.g.), (ii) model group (MOD, D-galactose 250 mg/kg i.p.+0.9% saline, i.g.), (iii) Dihydroquercetin group (DHQ, D-galactose 250 mg/kg i.p.+dihydroquercetin 9 mg/kg, i.g.), and (iv) Donepezil group (DON, D-galactose 250 mg/kg i.p.+donepezil 1.35 mg/kg, i.g.). With the exception of the control group, all rats underwent intraperitoneal injection of D-galactose (250 mg/kg) daily to induce ageing rat models, as evidenced by literature (Aydın et al., 2016; Fasina et al., 2022; Ibrahim et al., 2019; Mathimaran et al., 2021), with minor modifications. Meanwhile, the control group subjected to equivalent volume of saline. Subsequent to each modelling procedure, rats in the various treatment groups were given the corresponding drugs orally, while equal amounts of the vehicle were administered to rats in the CON and MOD groups. The dosage of dihydroquercetin was determined on the basis of the recommended daily intake of ≤100 mg/day for adults, as established by China’s National Health Commission (National Health Commission of the People’s Republic of China, 2021). Meanwhile, 15 mg/day of donepezil was designated as the adult dosage. The body surface area (BSA) normalization method was utilised for the conversion of doses across species. Body weight was measured at 2-week intervals for each rat throughout the duration of the treatment. The experiment was conducted over a period of 12 consecutive weeks.

### Ethics approval and consent to participate

The experimental protocol involving the use of animals was conducted in accordance with the guidelines of the Animal Experiments Committee, and was approved by the Animal Ethics Committee of China Technology Industry Holdings (Shenzhen) Co., Ltd. (approval NO.202300167). All methodologies employed in this study conformed to the established guidelines and regulations.

### Quantitative scoring for rat physical signs

As stated in the “Clinic terminology of traditional Chinese medical diagnosis and treatment-Part 2: Syndromes/patterns (GB/T 16,751.2-2021)”, in conjunction with the extant literature (Cui et al., 2014; Li D et al., 2025), a quantitative scoring table for rat physical signs was formulated, as illustrated in Table 1. Quantitative scoring was conducted for the physical signs of each group of rats. Each scoring was carried out independently by two trained researchers who were blinded to the group allocations (CON, MOD, DHQ, DON). A physical evaluation of the rats was conducted, and the results were documented. The mean score of the two operators was then taken as the final score for that physical sign.

**TABLE 1:** Quantitative scoring form for the TCM physical signs of rats.

Main symptoms	Nornal (score: 0)	Mild (score: 1)	Moderate (score: 2)	Severe (score: 3)
Coat condition	Smooth and glossy coat	Dull hair on the back	Dry and lusterless hair on the back	Dry, ruffled, and reduced hair over the whole body
Skin elasticity	Good skin elasticity	Slightly reduced skin elasticity	Loose skin	Poor skin elasticity, easily stretched
Activity and fatigue	Normal activity	Reduced activity	Tired, prefers lying down	Lies still, moves clumsily and slowly
Resistance upon capture	Strong resistance	Moderate resistance	Weak resistance	Minimal or no resistance
Huddling behavior	No huddling	Occasional huddling	Frequent huddling	Huddling with reduced movement

### Morris Water Maze (MWM) test

**FIGURE 2:**
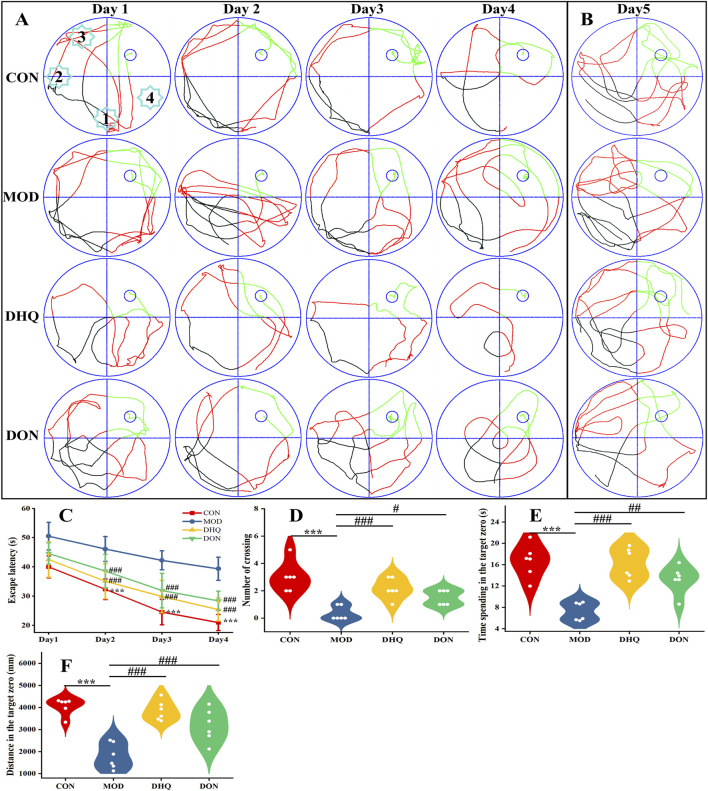
DHQ improved learning and memory function in D-galactose-induced cognitive impairment rats. The directional navigation test across days 1 through 4 **(A)**, the spatial probe test on the fifth day **(B)**, the escape latency time in directional navigation test **(C)**, the number of platform crossings in the spatial probe test **(D)**, the time spent in the target quadrant in the spatial probe test **(E)**, and the total distance travelled in the target quadrant in the spatial probe test **(F)**. Data represented as mean ± S.D. (n = 6).

The MVW test is a research instrument that assesses the spatial orientation capabilities of rats, thereby serving as a fundamental indicator in studies pertaining to learning and memory. The entire MWM test was conducted over 5 days. During the course of the MWM test, rigorous blinding procedures were consistently implemented throughout the data acquisition process, with the tracking data were conducted by an independent researcher who was unaware of the group allocations. Prior to the MWM test, all rats were subjected to an adaptive training regime. Subsequently, all rats were subjected to a directional navigation test spanning across days 1 through 4. Each rat was trained four times daily, entering the water randomly from one of four entry points (illustrated Figure 2A), with a maximum time allowance of 60 s to swim. If a rat successfully reaching a platform within 60 s, escape latency, defined as the time taken from entry to climbing onto the platform, was documented. If the rat was unable to locate the platform, the escape latency was documented as 60 s, after which the rat was navigated towards the platform and remained there for 10 s. The final day of the trial was designated for a spatial probe test. On the fifth day, the platform was removed, and the rats were released into the water from the quadrant opposite the original platform location. The observations encompassed metrics such as the number of crossings over the original platform area, the duration spent in that quadrant, and the trajectories of their swimming.

### Sample collection

Following the completion of behavioural testing, all experimental rats were anaesthetised using the inhaled anaesthetic isoflurane. Blood samples were extracted and subjected to a centrifugation process at 3,500 rpm for a duration of 10 min. The resultant serum was subsequently separated and stored at −80 °C for subsequent analysis. The heart, liver, spleen, and thymus were collected and weighed, after which the viscera indexes were calculated. The brain tissue was collected and subjected to a rinsing process with pre-cooled 0.9% saline solution. For pathomorphological analysis, a portion of rat brain tissue samples were fixed within 4% paraformaldehyde for subsequent analysis. Moreover, the remaining part of the rat brain tissue samples were separated into cortex and hippocampus tissues and preserved in liquid nitrogen for subsequent analysis.

### Hematoxylin-eosin (HE) staining

The brain tissue was preserved by fixation in 4% paraformaldehyde, then embedded, sectioned, and finally stained with hematoxylin and eosin. The pathological changes to the brain tissue were observed via SQS-40P slide scanner system (Teksqray, Shenzhen, China).

### Nissl staining

Nissl staining was utilised to quantify neuronal loss. The brain slices were subjected to Nissl staining for 10 min, followed by a thorough rinsing process involving two rinses. Thereafter, the slices were dehydrated using a gradient ethanol series, subsequently transparentised with xylene, and sealed with neutral gum. For quantitative analysis, Nissl-positive neurons in the CA1, CA3, and DG regions of the hippocampus were counted under light microscopy. Neurons that exhibited a discernible nucleus and clearly distinguishable cytoplasmic Nissl bodies were defined as positive neurons. Within each hippocampal subregion on each section, two fields of view were randomly selected. The mean count per field was used for statistical analysis.

### Enzyme-linked immunosorbent assay (ELISA)

The levels of inflammatory factors including TNF-α, IL-1β and IL-6 were detected in the hippocampus of rats according to the instructions of the ELISA kit.

### Western blot analysis

In the present investigation, the total protein content of the hippocampus was extracted using RIPA lysis buffer and subsequently quantitated using a BCA protein assay kit. Samples of equivalent protein quantity underwent separation by SDS-PAGE gel, followed by electrophoresis and subsequent transferring to a PVDF membrane. Subsequent to blocking of the nonspecific binding sites on the membranes with 5% skim milk, the primary antibodies, including TLR4 (1:6000), NF-κB p65 (1:6000), phospho-NF-κB p65 (1:1000), IL-1β (1:1000), CASP1 (1:6000), NLRP3 (1:1000), BAX (1:6000), BCL2 (1:3000), and CASP3 (1:6000), were added and incubated at 4 °C overnight. Thereafter, the membranes were subjected to three washes with TBST, and then incubated with HRP-conjugated secondary antibodies at room temperature for 1 h. Finally, the protein bands were detected via and ECL method, and subsequent analysis via ImageJ software.

### Statistical analysis

The data were expressed as the mean value ± standard deviation (S.D.). Repeated-measures analysis of variance (RM-ANOVA) was utilised to evaluate the escape latency data from day 1 to day 4 in the directional navigation test. Other differences between the groups were assessed with one-way analysis of variance (ANOVA). The probability value of *p* < 0.05 was utilised to ascertain the statistical significance of the results. Statistical significance was set at ***p < 0.001, **p < 0.01, *p < 0.05 vs. CON group; ###*p* < 0.001, ##*p* < 0.01, #*p* < 0.05 vs. MOD group.

### Molecular docking

A molecular docking investigation was conducted to examine the interaction between dihydroquercetin and hub targets in apoptosis and TLR4/NF-κB/NLRP3/CASP1 signaling pathways, including BAX, BCL2, CASP3, TLR4, IKKA, IKKB, NFKB1, NLRP3, ASC, CASP1, and IL-1β. The chemical structure of dihydroquercetin was obtained from the TCMSP database (https://old.tcmsp-e.com/tcmsp.php), and saved as a ligand in mol2 format. The 3D crystal structures of aforementioned targets, retrieved from the Protein Data Bank database (http://www.rcsb.org/pdb), underwent a series of preprocessing, optimization, and minimization procedures, ultimately being stored as receptors. Schrödinger Maestro software suite (version 11.1, Schrödinger, L.L.C.) was utilised for the molecular docking analysis. A comprehensive evaluation of the docking score, binding energy and the creation of hydrogen bonds in the ligand-receptor complex was conducted to determine the definitive stable conformation.

## Results

### Dihydroquercetin improve physical sign in D-galactose-induced aging rat model

As demonstrated by visual observations, rats in the model group exhibited pronounced aging phenotypes, including typical symptoms such as dry and sparse hair, reduced hair density, impaired skin elasticity, mental dullness, prolonged immobility, sluggish response, and decreased locomotor activity. As the modelling period progressed, there was a marked exacerbation of the symptoms related to the ageing process. Subsequent to week 8, the quantitative physical sign scores of the model group were found to be significantly higher than those of the control group. Conversely, rats administered dihydroquercetin exhibited a substantial alleviation of age-related symptoms (Figure 1B). It is evident that donepezil was less efficacious than dihydroquercetin in ameliorating ageing phenotypes. Throughout the experiment, no animals perished as a consequence of treatment with D-galactose, dihydroquercetin and donepezil.

As demonstrated in Figure 1C, the body weight of the rats in each group exhibited a consistent increase over time. However, rats in the donepezil group exhibited a slower rate of weight gain than the model and control groups. No statistically significant disparities were observed in body weight among the control, model, and dihydroquercetin cohorts.

As illustrated in Figure 1D, the induction of D-galactose resulted in a significant decrease in heart, liver, spleen, and thymus indexes, in comparison to the control group. Supplementation with dihydroquercetin elicited a substantial elevation.

### Dihydroquercetin ameliorated D-galactose-induced cognitive impairment

To further investigate the effects of dihydroquercetin on the spatial learning and memory function, the MWM tests were performed. As demonstrated in Figure 2A, in the initial phase of the directional navigation test, no statistically significant variations in escape latency were observed among the entire cohort of rats. As the training days progressed, a gradual decline in the escape latency of the rats was observed. Notably, the rats in the model group exhibited an increased escape latency in comparison with the control group. This finding suggests that the spatial learning ability of rats treated with D-galactose was reduced. In contrast, comparatively reduced escape latency was observed in rats in dihydroquercetin group as well as in donepezil group (Figure 2C). In the ensuing spatial probe trial conducted on the fifth day, the rats within the model group exhibited a general decline in performance, as evidenced by a decrease in the number of platform crossings, a reduction in the time spent in the target quadrant, and a decrease in the total distance travelled in that quadrant, when compared to the control group. Furthermore, the aforementioned behaviour exhibited a marked enhancement following treatment with dihydroquercetin, with a substantial escalation in the number of platform crossings (6.58-folds), the time spent in the target quadrant (2.25-folds), and the total distance travelled in the target quadrant (2.14-folds), respectively (Figures 2B,D–F).

### Dihydroquercetin mitigated D-galactose-induced neuronal injury in hippocampus

**FIGURE 3:**
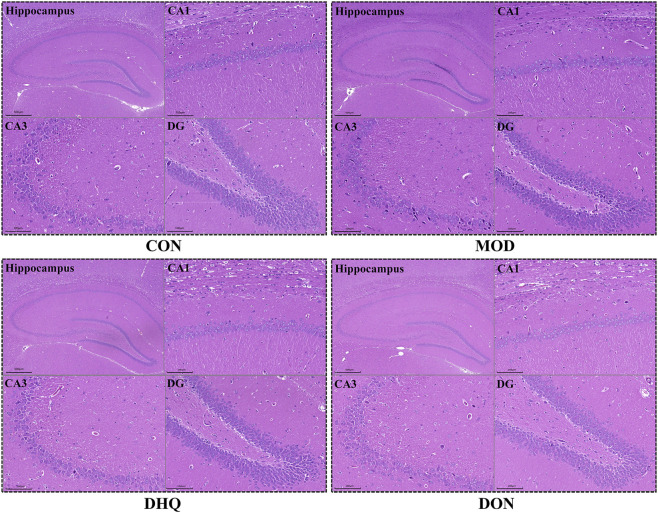
Neuroprotection effect of DHQ in hippocampus’ CA1, CA3 and DG regions (HE staining, scale bar = 100 μm).

The examination of histopathological changes was conducted using HE staining, with a focus on the hippocampus, a region susceptible to damage from early cognitive impairment. The HE staining revealed that the neurons in the hippocampal region of the control group exhibited a well-arranged structure, with normal-appearing nuclei and abundant cytoplasm. By contrast, the model group demonstrated disordered cell arrangement and a variable size of neural cells, accompanied by a marked reduction in the number of neurons. Supplementation with dihydroquercetin has been demonstrated to alleviate the neuronal histological damage in the hippocampus caused by D-galactose, and to reduce neuronal loss (Figure 3).

**FIGURE 4:**
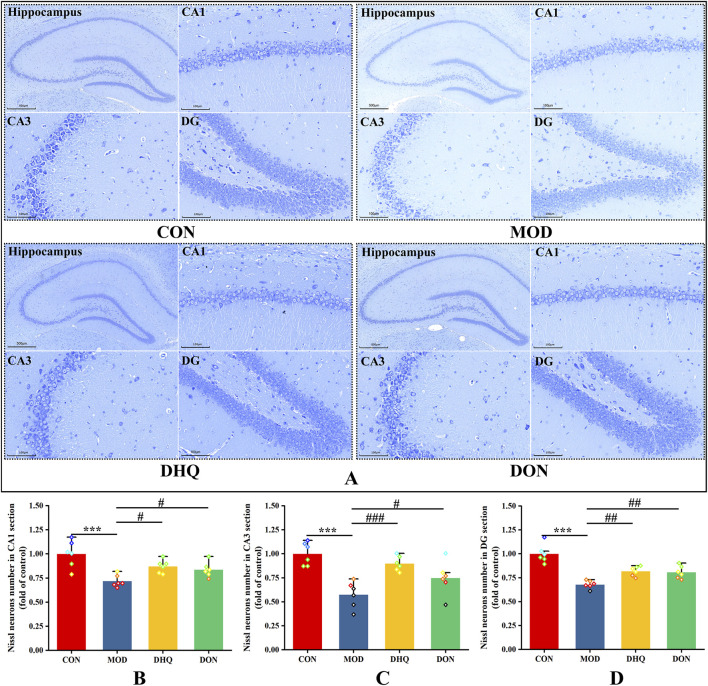
Dihydroquercetin mitigated neuronal injury in hippocampus. Nissl staining in the CA1, CA3 and DG regions of hippocampus (**(A)**, scale bar = 100 μm), the number of Nissl-positive neurons in CA1 section **(B)**, the number of Nissl-positive neurons in CA3 section **(C)**, the number of Nissl-positive neurons in DG section **(D)**. Data represented as mean ± S.D. of three rats, with two random areas from each sample.

Nissl staining (Figure 4A) served to further validate these observations. The neurons in the control group exhibited normal characteristics with regular, densely stained Nissl bodies. Conversely, D-galactose-induced neuronal atrophy in the model rat manifested as a decline in Nissl bodies and disruption of nuclear integrity. Furthermore, a substantial decrease in the number of Nissl-positive neurons was detected in the hippocampus' CA1, CA3, and DG regions in model rats, in comparison to the control group. It is worthy of note that, supplementation with dihydroquercetin treatment induced a substantial increase in the number of neurons. The quantification of these results is demonstrated in Figures 4B–D.

### Dihydroquercetin inhibited D-galactose-induced neuroinflammation in hippocampus

**FIGURE 5:**
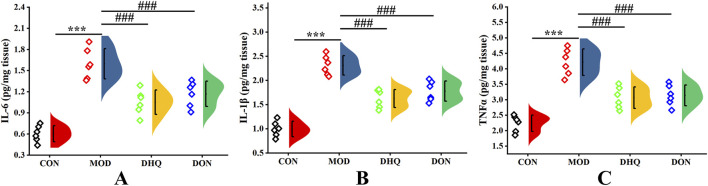
Dihydroquercetin inhibited neuroinflammation in hippocampus. The expression of IL-6 **(A)**, the expression of IL-1β **(B)**, and the expression of TNF-α **(C)** in hippocampal region. Data represented as mean ± S.D. (n = 6).

The release of neuroinflammatory cytokines is a salient feature in the development of cognitive impairment. As illustrated in Figure 5, D-galactose intervention led to a substantial increase in the levels of IL-6, IL-1β, and TNF-α in the model rat brain. Supplementation with dihydroquercetin induced a significant reduction the secretion of IL-6, IL-1β, and TNF-α in the brain, with a decrease of 34.37%, 29.44%, and 24.70%, respectively.

### Dihydroquercetin inhibited D-galactose-induced neuronal apoptosis in hippocampus

**FIGURE 6:**
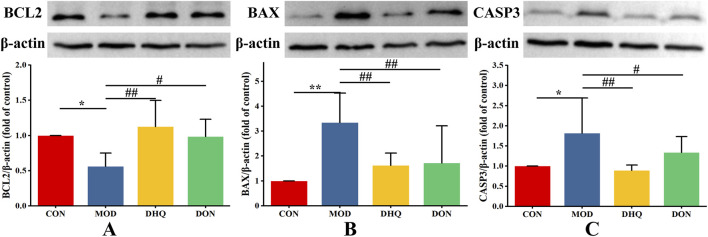
Protective effect of DHQ against apoptosis in hippocampus. Western blot analysis of BCL2 **(A)**, BAX **(B)**, and CASP3 **(C)** in hippocampal region. Data represented as mean ± S.D. (n = 4).

To further investigate the protective effect of dihydroquercetin against neuronal apoptosis caused by D-galactose, the expressions of important markers, including anti-apoptotic marker (BCL2) and pro-apoptotic markers (BAX, CASP3), were determined by Western blot analysis. As illustrated in Figures 6A–C, D-galactose administration resulted in a substantial augmentation in BAX and CASP3, concomitant with a conspicuous diminution in BCL2 within the hippocampus. In contrast, these changes were evidently alleviated by dihydroquercetin treatment. Specifically, the suppression of BAX, CASP3 expression and the enhancement of BCL2 expression by dihydroquercetin led to a reduction of the BAX/BCL2 ratio. The results indicate that dihydroquercetin exerts an anti-apoptotic effect on neurons by reducing the BAX/BCL2 ratio and inhibiting CASP3 activity in the rat hippocampus.

### Dihydroquercetin attenuated D-galactose-induced pyroptosis through modulation of the TLR4/NF-κB/NLRP3/CASP1 signaling pathways in hippocampus

**FIGURE 7:**
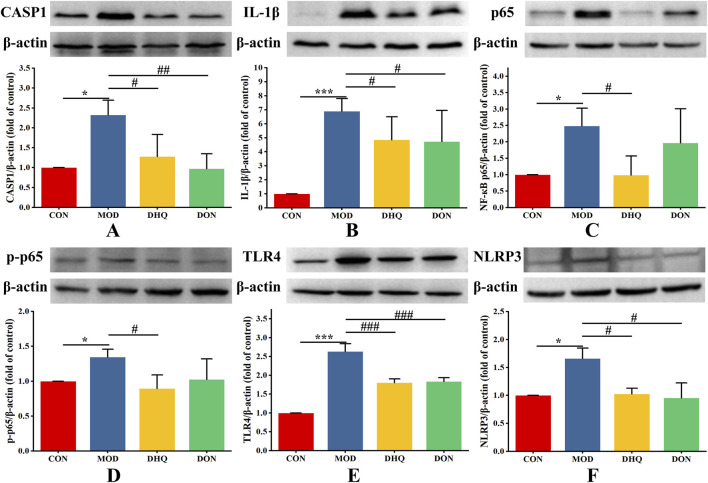
DHQ inhibits TLR4/NF-κB/NLRP3/CASP1 signaling pathway in hippocampus. Western blot analysis of CASP1 **(A)**, IL-1β **(B)**, NF-кB p65 **(C)**, p-NF-кB p65 **(D)**, TLR4 **(E)**, and NLRP3 **(F)**, in hippocampal region. Data represented as mean ± S.D. (n = 4).

In order to provide further confirmation of the underlying mechanism of dihydroquercetin in pyroptosis induced by D-galactose, the expressions of pyroptosis-related proteins were determined by Western blot analysis. As demonstrated in Figures 7A–F, the expressions of TLR4, NF-кB, p-NF-кB, IL-1β, NLRP3, and CASP1, were significantly increased in the hippocampus of model rats induced by D-galactose (vs. CON, *p* < 0.05). In contrast, supplementation with dihydroquercetin effectively reduce the expressions of these factors (vs. MOD, *p* < 0.05). Consequently, the aforementioned results verified that dihydroquercetin attenuated D-galactose-induced pyroptosis by suppressing the TLR4/NF-κB/NLRP3/CASP1 signaling pathway.

Critically, as a first-line medication for Alzheimer’s disease in clinical practice, a substantial body of evidence supports that donepezil not only exhibits potent acetylcholinesterase inhibitory activity, but also exerts significant anti-inflammatory and anti-pyroptotic effects (Kim et al., 2021; Youn et al., 2024). The superior efficacy observed in the present study for DHQ in comparison with donepezil suggests that the multi-targeted action of DHQ on both apoptosis and pyroptosis results in a more potent neuroprotective effect.

### Molecular docking analysis

**FIGURE 8:**
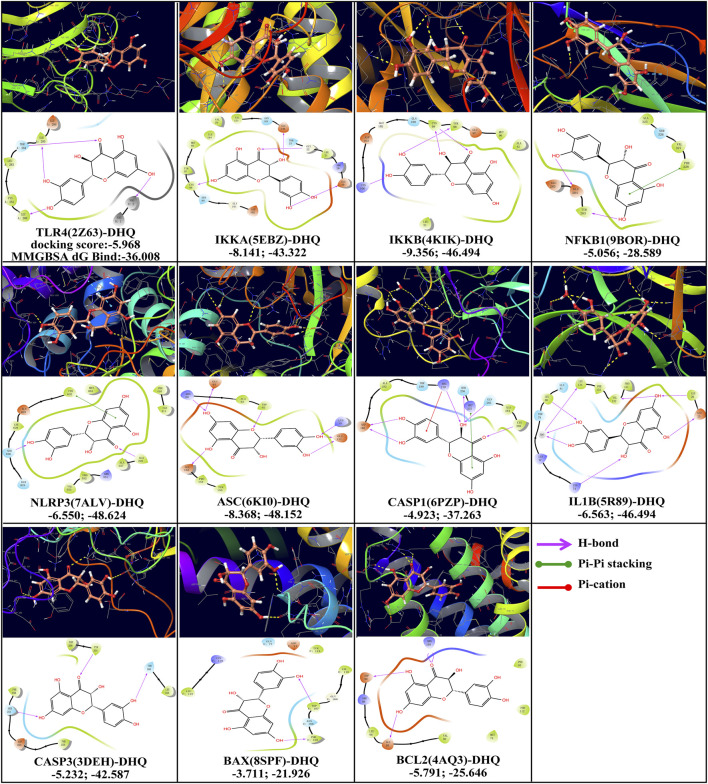
Molecular docking analysis of DHQ interacting with target proteins in 3D and 2D diagrams.

As demonstrated in Figure 8, the docking scores, binding energy and molecular docking models between dihydroquercetin and hub targets, including TLR4 (PDBID: 2Z63), IKKα (5EBZ), IKKβ (4KIK), NFKB1 (9BOR), NLRP3 (7ALV), ASC (6KI0), CASP1 (6PZP), IL1B (5R89), BAX (8SPF), BCL2 (4AQ3), and CASP3 (3DEH), were illustrated. Dihydroquercetin exhibited a comparatively low binding score and low binding energy during docking with all the test targets, suggesting potential binding affinity. In particular, the docking scores of IKKα-DHQ, IKKβ-DHQ, and ASC-DHQ, exhibited values lower than −7.00, suggesting enhanced stability during the binding process. Observations demonstrated that the benzene ring structures and phenolic hydroxyl groups of the A and B rings of dihydroquercetin, as well as the carbonyl structure of the C ring, are capable of forming a variety of intermolecular forces with the target amino acid residues, encompassing H-bonds, Pi-Pi stacking, and Pi-cation interactions. For instance, the amino acid residues LEU 280 and ILE 285 of TLR4 on chain A were observed to form hydrogen bond interactions with the phenolic hydroxyl group of A and B rings on dihydroquercetin, while the amino acid residues THR 284 hydrogen bonded with the carbonyl of C ring on dihydroquercetin. The amino acid residues TYR 632 of NRLP3 form Pi-Pi stacking interactions with the benzene ring (A ring) on dihydroquercetin. In addition, dihydroquercetin’s benzene ring (B ring) formed Pi-cation with the amino acid residues ARG 179 of CASP1.

## Discussion

Aging is an intricate biological phenomenon defined by progressive deterioration of cellular homeostasis, particularly within the central nervous system (CNS). This decline manifests as a significant acceleration in neuronal loss, driven by programmed cell death (PCD) mechanisms, which ultimately results in cognitive impairment (Salter and Stevens, 2017; Huang et al., 2018; Wan et al., 2020). As a recently identified form of PCD involving inflammation, pyroptosis has been demonstrated to contribute to neurodegeneration through the elevation of inflammatory cytokines, such as IL-1β and IL-18, serving as a primary trigger of neuronal demise (Moujalled et al., 2021). Therapeutic strategies that target the pyroptosis signalling pathway have been shown to be efficacious, with evidence indicating that the inhibition of NLRP3 inflammasomes, caspase-1, or gasdermin D can attenuate Alzheimer’s disease-related pathological features (Tang, 2025). The present study demonstrated, for the first time, that dihydroquercetin supplementation significantly ameliorated D-galactose-induced cognitive impairment in ageing rats, with its effects being associated with the modulation of markers related to the concurrent and coordinated attenuation of two pivotal programmed cell death pathways: apoptosis and pyroptosis, in hippocampus. By contrast with earlier investigations reporting DHQ’s anti-apoptotic or anti-pyroptosis effects within distinct models, such as rotenone-induced Parkinsonism, alcoholic liver steatosis or diabetic nephropathy, operating within isolated pathways, our study provides integrated evidence that DHQ treatment not only associates with the priming (via TLR4/NF-κB) and activation (via NLRP3/CASP1/IL-1β) of pyroptosis, but also corrects the imbalance of mitochondrial apoptotic regulators (BCL2/BAX/CASP3), within a rat model of ageing and cognitive impairment. These findings suggest that DHQ has the potential to be a promising therapeutic candidate for age-related cognitive decline, with its association with dual modulation of apoptosis and pyroptosis, representing a novel pharmacological approach.

The TLR4/NF-κB axis serves as a pivotal upstream regulator of both neuroinflammation and programmed cell death pathways. The results of the present study demonstrate that DHQ supplementation was associated with significant suppression of TLR4 and phospho-NF-κB expression, which is consistent with an interruption of neuroinflammatory priming. This observed inhibition coincided with reduced NLRP3 inflammasome activation, a critical step in pyroptosis execution. Subsequently, levels of cleaved CASP1 decline, accompanied by a reduction in IL-1β maturation, a process linked to gasdermin D (GSDMD)-mediated pore formation, which is a hallmark of pyroptotic cell death. These findings are consistent with the documented anti-inflammatory effects of DHQ (Akinmoladun et al., 2022; Ding et al., 2018). Nevertheless, the present study is the first to report an association between DHQ supplementation and the suppression of neuronal pyroptotic cascades *in vivo*. Crucially, DHQ administration was simultaneously linked to modulation of apoptotic pathways, as indicated by elevated BCL2 expression, suppressed BAX, and reduced CASP3 activation. The resultant stabilization of mitochondrial integrity may contribute to the prevention of cytochrome c release and apoptosome formation, thereby potentially preserving neuronal viability (Kawamoto et al., 2014; Li T et al., 2025). This coordinated association with markers of both pyroptosis and apoptosis could help explain the observed efficacy of DHQ in preserving neuronal structure and function.

The process of chronic neuroinflammation and neuronal death creates a self-reinforcing “neurodegenerative loop.” Pro-inflammatory cytokines (e.g., IL1β) have been demonstrated to activate death receptors (e.g., TNFR1), thereby potentially initiating apoptosis (Sagulenko et al., 2013). In contrast, the release of damage-associated molecular patterns (DAMPs) by apoptotic cells serves to exacerbate microglial TLR4/NF-κB signalling, thereby amplifying NLRP3 inflammasome activation (Venegas et al., 2017). The present study demonstrates that DHQ treatment is associated with disruption of this vicious cycle at multiple nodes, as indicated by reduced TNF-α, IL-1β, and IL-6 release, inhibition of TLR4/NF-κB-dependent inflammasome priming, and suppression of NLRP3/CASP1-mediated pyroptosis markers.

Microglia, as the primary resident immune cells in the central nervous system (CNS), play a pivotal role in determining the neuroinflammatory environment. The pro-inflammatory M1 phenotype, instigated by DAMPs and TLR4 activation, is characterised by the robust production of several cytokines, including TNF-α, IL-1β and IL-6 (Tang and Le, 2016). The TLR4/NF-κB pathway acts as a key upstream regulator that primes the NLRP3 inflammasome, thereby promoting M1 polarization and creating a cycle of chronic neuroinflammation observed in ageing and neurodegenerative models (Ibrahim et al., 2023; Yang et al., 2020; Liu et al., 2020). Furthermore, the downstream execution of pyroptosis is intrinsically linked to microglial activation. The direct inhibition of TLR4 with TAK-242 in an APP/PS1 Alzheimer’s model has been shown to shift microglial polarization from M1 to M2, concurrently inhibiting the MyD88/NF-κB/NLRP3 pathway and improving cognitive function (Cui et al., 2020). In a similar manner, in a D-galactose/AlCl_3_-induced AD-like model, festidinol improved outcomes in association with inhibition of the TLR4/NF-κB/NLRP3 axis, a pathway that is intimately linked to microglial-driven inflammation (Wongpun et al., 2023). Therefore, the observed downregulation of the TLR4/NF-κB/NLRP3/CASP1 pathway and pro-inflammatory cytokines by DHQ suggests a possible modulation of microglial activity and a potential shift away from the pro-inflammatory M1 state.

The synergy between DHQ’s reported antioxidant and the observed anti-inflammatory properties may further strengthens its neuroprotective profile. D-galactose has been demonstrated to generate reactive oxygen species (ROS), which can activate the NF-κB pathway and promote NLRP3 assembly (Ali et al., 2021; Wongpun et al., 2023). As demonstrated in other models, DHQ’s catechol structure facilitates efficient ROS scavenging, a process which resulted in a reduction of lipid peroxidation and an enhancement of glutathione synthesis (Akinmoladun et al., 2022). This places DHQ at the intersection of oxidative stress, inflammation, and PCD, thus offering a comprehensive neuroprotective strategy that is distinct from monofunctional agents.

Furthermore, a critical consideration for centrally acting therapeutic candidates is their ability to reach the brain at sufficient concentrations to exert biological effects. Despite the fact that the present study did not directly measure DHQ concentrations in the hippocampus, converging evidence from the extant literature and our potent pharmacodynamic outcomes provide strong support for the dosage selection that orally administered DHQ at 9 mg/kg can achieve functionally relevant levels in the brain. It is acknowledged that DHQ, like many flavonoids, has limited permeability across the blood-brain barrier (BBB) (Inoue et al., 2019). However, its neuroprotective efficacy in various neurodegenerative models across a range of doses indicates effective central engagement. Notably, in a cerebral amyloid angiopathy model, dietary supplementation with 3% DHQ resulted in pleiotropic neuroprotection, including reduced Aβ production and suppressed neuroinflammation (Inoue et al., 2019). Furthermore, in a rotenone-induced Parkinsonism model, DHQ, administered at doses ranging from 0.25 to 1.0 mg/kg (i.p.), demonstrated a positive effect on outcomes by modulating the NF-κB-mediated inflammatory pathway in the brain (Akinmoladun et al., 2022). In another Parkinson’s disease model utilizing LPS or 6-OHDA, the intragastric administration of DHQ at a dose of 5–15 mg/kg effectively protected dopaminergic neurons and inhibited microglial activation (Yang et al., 2024). It was observed that even an intravenous administration of an extremely low dose (0.5–2 μg/kg) of DHQ was effective in counteracting LPS-induced neuroinflammation and memory deficit (Alam and Krishnamurthy, 2022). The extant literature suggests that even limited brain penetration of DHQ, at the dose of 9 mg/kg (administered orally), is adequate to modulate the intricate neuroinflammatory and cell death pathways in the hippocampus.

## Conclusion

The present study identifies DHQ as a novel food resource associated with multitarget modulation of neuronal apoptosis and pyroptosis markers as well as demonstrating its efficacy in mitigating D-gal-induced cognitive impairment in conjunction with the suppression of the TLR4/NF-κB/NLRP3/CASP1 axis. The disruption of crosstalk between neuroinflammation and PCD represents a significant correlate of DHQ supplementation, resulting in neuroprotection. The natural origin of DHQ, in conjunction with its association with the dual modulation of apoptosis and pyroptosis pathways within the ageing hippocampus, positions it as a promising multi-target candidate for mitigating age-related cognitive decline.

## Data Availability

The original contributions presented in the study are included in the article/supplementary material, further inquiries can be directed to the corresponding authors.
